# Towards sustainable management of forest residues in the southern Apennine Mediterranean mountain forests: a scenario-based approach

**DOI:** 10.1186/s13595-022-01128-w

**Published:** 2022-03-28

**Authors:** Maria Teresa Pergola, Luigi Saulino, Maria Castellaneta, Angelo Rita, Giovanni Pecora, Mario Cozzi, Nicola Moretti, Osvaldo Pericolo, Domenico Pierangeli, Severino Romano, Mauro Viccaro, Francesco Ripullone

**Affiliations:** 1 Ages s.r.l. s - Spin-off Accademico, Università degli Studi della Basilicata, Viale dell’Ateneo Lucano, 10 – 85100 Potenza, Italy; 2 Dipartimento di Agraria, Università di Napoli Federico II, via Università 100, IT-80055 Portici (Napoli), Italy; 3 Scuola di Scienze Agrarie, Forestali, Alimentari ed Ambientali, Università di Basilicata, viale dell’Ateneo Lucano, 10. I-85100 Potenza, Italy

**Keywords:** Forest management plans, Woody residues, Soil organic content (SOC), Soil organic carbon stock (SOCS), Soil fertility

## Abstract

**Key message:**

Managing forest residues according to the carbon content of the soil helps to minimize the ecological footprint of their removal.

**Context:**

In Mediterranean mountain ecosystems, unsustainable harvesting of wood residues might contribute to land degradation, carbon, and nutrient depletion in forest soils.

**Aims:**

This study aimed to assess the amount of forest biomass residues that should be left on-site to minimize the depletion of soil fertility.

**Methods:**

We estimated the availability of biomass residues in the public forest land of the Basilicata region of Southern Italy by collecting stand-scale inventory attributes from forest management plans. Subsequently, we quantified the amount of forest biomass residue released by implementing a scenario-based approach.

**Results:**

Approximately 5800 m^3^ year^−1^ of forest residues could be potentially available for bio-based industries at the regional scale within the next 10 years. Such residues mainly belong to broadleaved forest types, having a high variability in their soil organic stock (228.5–705.8 Mg C ha^−1^) and altitudinally spanning from 400 to 1500 m a.s.l. In these forests, the simulated scenarios displayed a wide range of average harvestable residues from 2.5 to 5.5 m^3^ ha^−1^, containing approximately 1.1 to 2.1 Mg ha^−1^ of organic carbon.

**Conclusion:**

Our study suggests that forest management plans are a useful source of information to estimate the available forest biomass residues consistently. In southern Mediterranean mountain forests, the management of forest residues according to soil carbon content helps to minimize the environmental impact and increase their sustainability.

## Introduction

Biomass resources have attracted policy attention as one of the essential components in both bio-based industry and the energy consumption mix diversification of European countries (Ferranti 2014; European Commission 2017; Sikkema et al. 2021). For the transition towards a sustainable climate-neutral economy, there have been increasing efforts and incentives over the last decade in utilizing woody biomass in the circular bio-economy as a sustainable feedstock for bio-based industries and bioenergy as a replacement for conventional fossil-based materials (Scarlat et al. 2015; European Parliament 2021). Both products and energy obtained from woody biomass have the potential to mitigate significant environmental impacts. However, when used for bioenergy purposes, their combustion needs to be compensated by the capture and storage of carbon (C) to be sustainable.

Additionally, the reuse of agricultural and forest residues, that would otherwise be underutilized, has the potential to create jobs and income, especially in rural areas, and to provide local and sustainable feedstock energy for communities by decreasing their dependency on the international fuel market (Hall 2002; Ahtikoski et al. 2008; Saidur et al. 2011; Shabani et al. 2013; Viccaro et al. 2019).

Although woody biomass is generally considered a renewable bioresource alternative to both fossil-based products and fuels, the current debate on biomass utilization critically evaluates its C neutrality and sustainability (Mather-Gratton et al. 2021). For instance, the lack of concentrated biomass resources and limited accessibility to mountain forests have hindered biomass supply chains. Biomass resources are usually spread over large and fragmented forest areas, agricultural activities, or dedicated coppice plantations (Verkerk et al. 2019; Viccaro et al. 2019; Saulino et al. 2019). Additionally, unfavorable climatic conditions, combined with the high biomass transportation costs, which account for up to 50% of the total delivery cost, and risks related to wood market instability and stochastic natural disturbances increase the uncertainty of forest biomass supply chains (Hall 2002; Flisberg et al. 2012; Shabani et al. 2013). Furthermore, the sustainability of bioenergy production from forests needs to be considered (Aguilar 2014; Leban et al. 2016), given that the production and use of wood to generate bio-based goods and energy has substantial environmental implications (Rafael et al. 2015; Erb et al. 2018). Currently, there is a highly debated trade-off between using forest biomass residues as bio-products to mitigate the environmental impact of fossil-based products and their on-site release to sustain and conserve the productivity and fertility of forest stands (Achat et al. 2015; Titus et al. 2021). This becomes relevant in Mediterranean forest ecosystems that are markedly vulnerable to soil degradation and soil organic carbon (SOC) fertilization, often associated with erosive processes (Lavee et al. 1998; Muñoz-Rojas et al. 2016), due to extensive land-use changes over time (Anaya-Romero et al. 2011; Francaviglia et al. 2017).

In forest ecosystems, the constant addition of decaying tree residues might represent the primary source of SOC and nutrients to the soil (Conforti et al. 2016), which are utilized by trees for their regeneration and establishment processes (Harmon et al. 1986; Motta et al. 2006). Conversely, the increasing removal of forest residues reduces the pool of C stored in dead organic matter and litter input to soil (Johnson and Curtis 2001; Eriksson et al. 2007; Vanhala et al. 2013), significantly decreasing the C and nutrient levels in forest soil. Against this degradation factor, the in situ release of biomass residues could provide a range of environmental benefits by simultaneously increasing the structure and species diversity of forest stands, microbiological and physiochemical properties, soil fertility, nutrient availability, and water retention in forest soil (Ali 2019; Manolis et al. 2019; Bonanomi et al. 2021). Research studies indicating the releasable amount of forest residues are mainly conducted in northern latitude forest ecosystems, in which species composition and soil properties are markedly different from those in Mediterranean environments. In Swedish forests, the suggested amount of wood ash to be added as a soil supplement to compensate for the loss of nutrients varies between 0.8 and 2.2 ton ha^−1^ per rotation period, depending on the region and local conditions (Börjesson 2000). For example, Norway spruce stands were reported to release approximately 15.0 kg dw ha^−1^ as the amount of fine fraction residues left on site post-harvest (Olsson et al. 1996; Wang et al. 2010). The southern USA considered that leaving 30% of logging residues on harvest sites is more than sufficient to meet the nutrient requirements (Perez-Verdin et al. 2009; Pokharel et al. 2017). However, according to the different US guidelines, the suggested proportion of residues left on the ground ranges from 10 to 33% of the harvestable biomass (Fletcher et al. 2011). Nevertheless, limited studies have evaluated the optimal amount of biomass residues that should be left on the soil to compensate for the removal of organic matter and essential macro-and micro-nutrients (Perez-Verdin et al. 2009), especially in the Mediterranean mountain environments. Releasing forest biomass residues on site in degraded Mediterranean forest ecosystems might enhance their productivity, soil stability, and vegetation recovery (Hueso-González et al. 2018).

Currently, forest biomass residues, such as logging residues, non-merchantable timber, and roundwood, are alternatively left or burnt on site, given that their collection is not economically viable for forest enterprises (Hueso-González et al. 2018; Vance et al. 2018). Currently, forestry residues are considered a suitable renewable bioresource alternative to fossil-based bioproducts (Perea-Moreno et al. 2017; Briones-Hidrovo et al. 2021) and potentially contribute to the ecological transition of the European Union (COM 2020). European countries have long treated the development of renewable sources as a priority in their energy policy and the promotion of energy efficiency (COM 2021). In light of the current COVID-19 pandemic, European countries have prioritized the transition towards sustainability (Köhler et al. 2019) by activating a series of extraordinary measures and financial resources to cope with the economic crisis triggered by the spread of the virus. On a national scale, the transition to bio-economy has reopened the attention on forests as a feedstock source for bio-based industries and bioenergy. However, the overall debate over sustainability transitions needs to consider both places and bio-resource types where transitions occur, which represent relevant factors in minimizing the ecological footprint of the bio-based economy sector.

Within this framework, this study aimed to explore the sustainability of biomass residue management following a set of on-site release scenarios of forest biomass residues in the public forest lands of the Basilicata region (Southern Italy). This is to supply regional and local administrations with quantitative information that could help support and promote the industry sector powered by biomass, preserving the functioning of forest ecosystems with priority.

## Materials and methods

### Study site

**Fig. 1:**
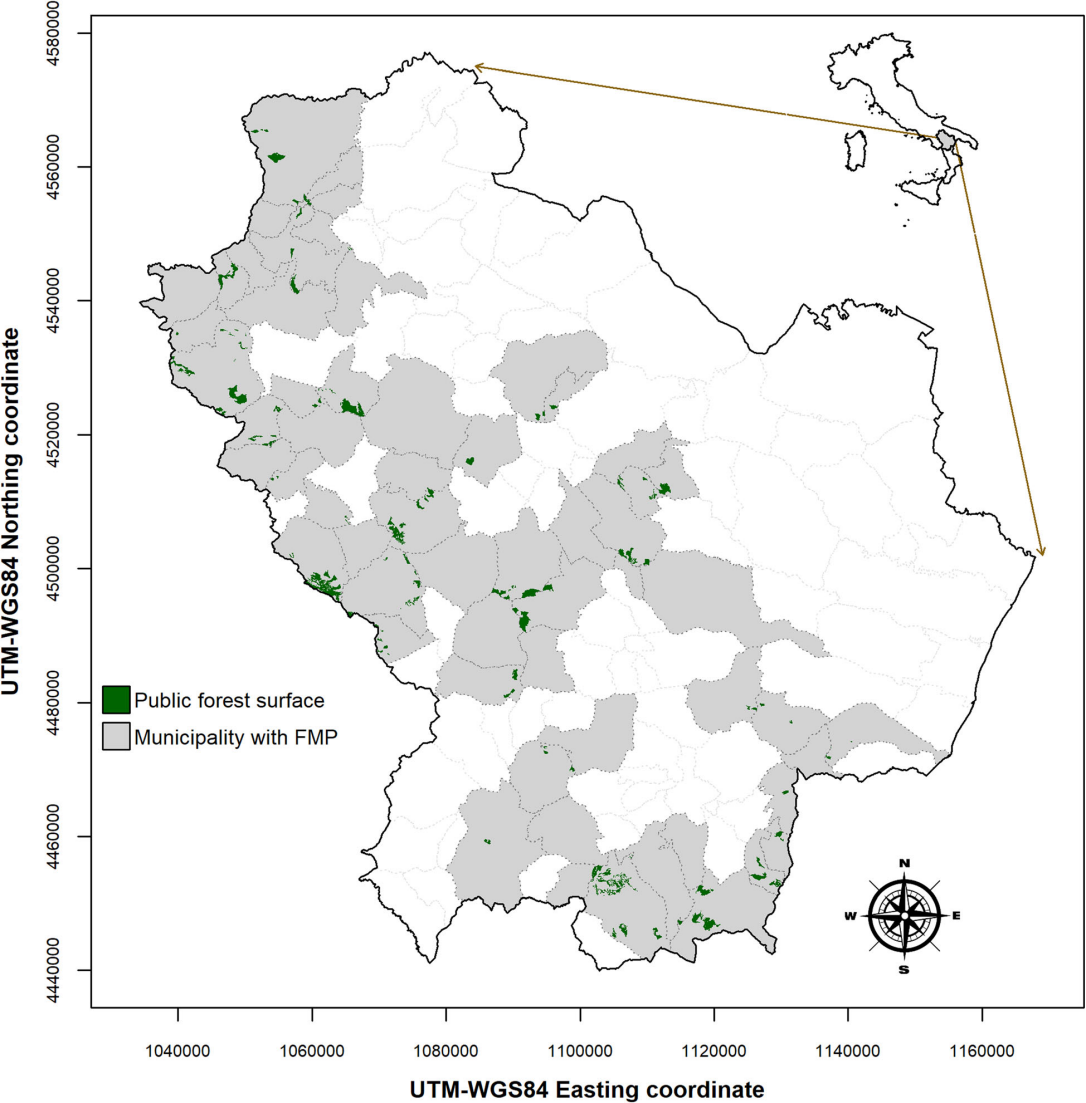
Spatial distribution of public forest surface cover (green polygons) under the management plan. Gray polygons indicated municipalities with forest management plan (FMP) document in force. In white municipalities without forest surface or with expired FMP

Forests cover approximately 35% (356,426 ha) of the overall administrative territory of the Basilicata region (Fig. 1). Such forest surfaces are spatially fragmented and distributed within a broad altitude range, from approximately 300 to 1700 m a.s.l. Less than 50% of these surfaces are public property and are owned and managed by land administration agencies (i.e., municipalities, national, and provincial park institutions). At the same time, the remaining forests are private properties owned by citizens or families rather than private companies. The size of forest surfaces varies according to property ownership, with public forests consistently over 50 ha. In these forests, the dominant vegetation categories are represented by broadleaved forests, with an evident prevalence of Mediterranean deciduous oaks covering 51.8% of the total forest area, followed by European beech (8.4%), Mediterranean evergreen oaks (7.9%), thermophilous shrublands (6.9%), other mesophilic and mesothermophilic broad-leaved forest stands (5.5%), riparian forests (3.9 %), chestnut forests (3.0%), and plantation forests with exotic tree species (0.6%) (Costantini et al. 2006). Conifer forests cover the remaining 12% of the forest area, of which subalpine and alpine conifers represent 3% and Mediterranean pine forests cover 9%.

### Collection of forest management plans and stand attributes

For each municipality, we acquired the forest management plans (FMP) in force for the public forest surface under a management plan. The FMP represents a pivotal document that provides forest resource inventory and spatiotemporal and quantitative management information at the forest parcel scale. The parcel serves as the smallest management unit of forest stands in which both ecological and silvicultural management criteria are implemented. From each FMP, we extracted an array of topographic and biometric attributes to quantitatively characterize public forest surfaces. At the parcel scale, the following inventory attributes were extracted: (i) surface area of the forest parcels (ha); (ii) the mean annual increment (m^3^ ha^−1^); and (iii) the aboveground standing volume (m^3^ ha^−1^). Moreover, the forest parcel polygons were used as spatial vector data. For each centroid of the forest parcel, the altitude value (m a.s.l.) was extracted from the freely available digital elevation model (DEM) at a resolution of 20 m (http://www.sinanet.isprambiente.it/it/sia-ispra/download-mais/dem20/view).

### Estimating the volume of forest residues

Commonly, the term “forest biomass residues” refers to woody biomass generated directly by forest management activities, such as logging residues, and by the wood processing industry, such as shavings, sawdust, and woodchips. Although biomass residues are generated from forest logging activities to the roundwood processing plant, in this study, we considered exclusively the biomass residues produced by on-site harvesting activities. Therefore, in our study, the term forest residues refers to the biomass residues directly resulting from the harvesting and processing activities of on-site logged trees. Such residues are defined as the primary class of forestry-derived biomass feedstock obtainable from forest harvesting, which is also indicated with the common terms “slash” or “brash” (Titus et al. 2021) and more specifically as fine wood debris (FWD; Camia et al. 2021).

Forest biomass residues mainly consist of upper stem and branch fractions with a diameter lower than 5 cm, excluding the stump, roots, and leaf biomass (except for conifers) of harvested trees. We quantified forest residues as a fraction of the harvestable aboveground standing volumes of each parcel of FMPs. Then, according to the species-specific percentage values estimated by Cozzi et al. (2013), for Basilicata forests, the volume of forest residues was estimated as 9 to 20% of total forest utilization (i.e., harvested aboveground standing volume, m^3^) of each parcel.

### Estimating the carbon content of biomass residues

To assess the organic C fraction potentially left on site for each forest parcel (*x*), the quantity of organic C content by forest residues (ROC, Mg ha^−1^) was calculated as follow:

$$ {\mathrm{ROC}}_x={\mathrm{AV}}_x\bullet \mathrm{c} $$

where, AV_*x*_ represents the aboveground residue volume (m^3^ ha^−1^) at parcel scale, and *c* is the conversion factor from volume to weight of organic C (Mg m^−3^). Since *c* depends on the tree species, for oak woods, beech woods, chestnut woods, hornbeam forests, other deciduous forests, and conifers, the species-specific *c* values were retrieved from Gasparini et al. 2013.

### Estimation of soil organic carbon stock (SOCS)

The SOC stock (Mg C ha^−1^) was calculated from the SOC content, bulk density, and coarse fragments at six depth intervals (0–5 cm, 5–15 cm, 15–30 cm, 30–60 cm, 60–100 cm, and 100–200 cm). Finally, the value of SOC at each parcel scale was obtained by summation of the organic contents of all six soil depth intervals. The stock of SOC data (OCSTHA in the SoilGrids database) was obtained from the recently released International Soil Reference and Information Center (ISRIC) (Hengl et al. 2017). The data available in a gridded format at a spatial resolution of 250 m were downloaded from the server ftp://ftp.soilgrids.org/data/recent.

### Scenarios of in situ released biomass residues

**Fig. 2:**
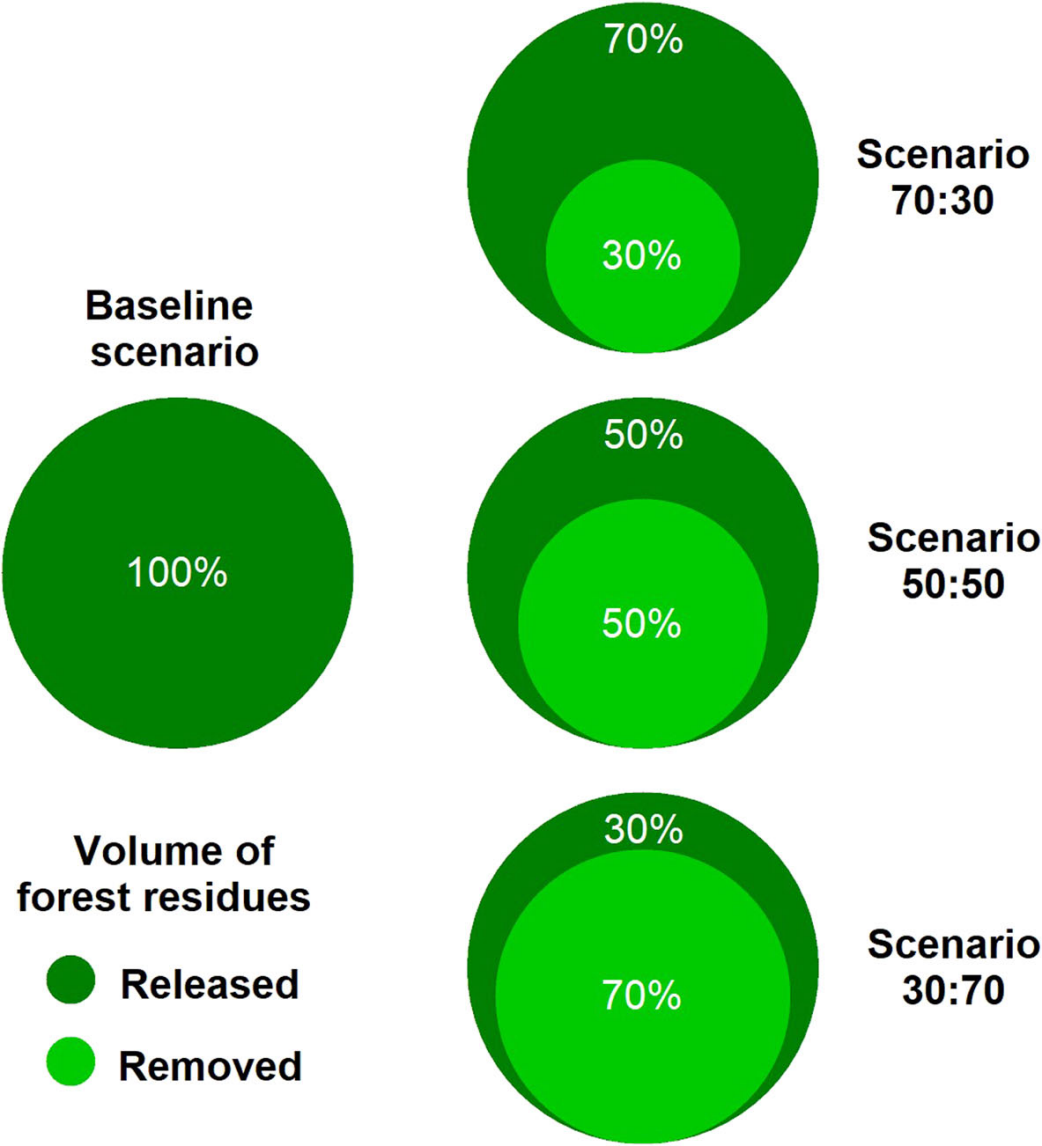
Scenarios simulated in the release of forest biomass residues. Each scenario was simulated taking into account the decrease in volume of forest residues released on the logging site. The baseline scenario corresponds to the release of 100% of the available forest residues. Decreasing scenarios consist in releasing on-site 70%, 50%, and 30% (dark green circles) of the available biomass residues by removing the respective reciprocal percentage residues fractions (light green circles)

To assess the potential availability of biomass residues for the Basilicata region, different biomass-releasing scenarios were simulated by computing them on the total available harvesting residues at the parcel scale (Fig. 2).

It was simulated from the release of 100% of the available forest residues as a baseline scenario. This scenario synthesizes a set of socio-economic, technological, and environmental circumstances, which justify the retention of the forest biomass residues at a logging site. Therefore, such a baseline scenario includes leaving all forest residues on site or processing them so that they are mulched or distributed on site for forest sustainable management goals, excluding any commercial and industrial use.

Three different scenarios were simulated by gradually decreasing the release of forest biomass residues from 100% to 70%, 50%, and 30%. These decreasing release scenarios consist of leaving a progressively lower quantity of biomass residues on forest sites, excluding them from commercial and industrial uses. These scenarios reflect increasing interest in the large-scale utilization of forest biomass residues for bio-based industry processes by quantifying their potential availability at a regional scale. Each scenario synthesizes a set of favorable socio-economic, technological, and environmental circumstances, allowing the removal of biomass residues from logging sites for commercial and industrial purposes. Moreover, the 30% scenario release represents the minimum benchmarking fraction of residues that should be left on site according to the guidelines for forest residue release in northern European countries (Titus et al. 2021). Therefore, we used 30% as a conservative threshold to minimize soil fertility loss in Mediterranean mountain ecosystem forests.

### Biomass residue sustainability ratio

For each simulated scenario, the sustainability of forest residue removal was evaluated by means of the ratio between the ROC and the existing SOC contents as follows:

$$ {\mathrm{RC}}_{\mathrm{Index}}=\frac{\mathrm{ROC}}{\mathrm{SOC}} $$

where RC_Index_ corresponds to the residue C index, and ROC and SOC correspond to the C content of the forest residues and soil, respectively. The range values of the dimensionless RC_Index_ are > 0; when its value is close to zero, the releases of forest residues on logging forest floor are low or null, while it assumes increasing values > 0 when the amount of forest residues on site increases.

### Data analysis

To identify the group of forest parcels with similar growth and productive attributes, an unsupervised K-means clustering analysis was performed (Hartigan and Wong 1979). The clusters of the forest parcel were partitioned by site and stand attributes: altitude, SOC, mean annual increment (MAI), and aboveground standing volume (ASV). The K-means algorithm allows identification of the cluster of forest parcels characterized by high intra-class similarity. The Euclidean distance method was used to assign each forest parcel to the closest cluster centroid. The optimal number of clusters was determined by the gap statistic (*k*) iterative method with 500 Monte Carlo bootstrapped samples, and by employing the first SE max criteria (with SE factor of 1) to identify the location of the maximum gap statistic value.

The contribution of each attribute to both dimensions was determined by principal component analysis (PCA). In the K-means partitioning clustering method, PCA is used to reduce the dimensions of the data. Each variable was considered significant when its percentage contribution exceeded the expected average contribution.

Management and analysis of the data were performed in R (R Core Team 2020) by means of “cluster” (Maechler et al. 2021), “FactoMineR” (Lê et al. 2008), and “factoextra” (Kassambara and Mundt 2020) packages.

## Results

### Quantitative characteristics of public forest stands

Currently, in Basilicata, there are 66 FMPs, 31 (47%) of which are expired and have not yet been renewed, and 35 (53%) of which are still enforced or recently expired. The forested surface area managed by FMPs accounts for 7680 hectares (2.2% of the entire forested area) with a total aboveground standing volume of 2.55 million m^3^. This standing volume mainly belongs to the productive high forests, although such volumes belong to coppice-managed stands on some public forest surfaces. In 62% of the municipalities, the forest parcels were managed for productive purposes, whereas the remaining 38% were alternatively managed for protective or recreational purposes.

Within the time windows of 10 years from 2018 to 2027, the available and potentially harvestable aboveground woody volume is 736,700 m^3^, whereas the overall volume of logging residues accounts for 50,800 m^3^, corresponding to 5800 m^3^ year^−1^ and 0.66 m^3^ ha^−1^ year^−1^ approximately.

### Clustered forest parcels

**Fig. 3:**
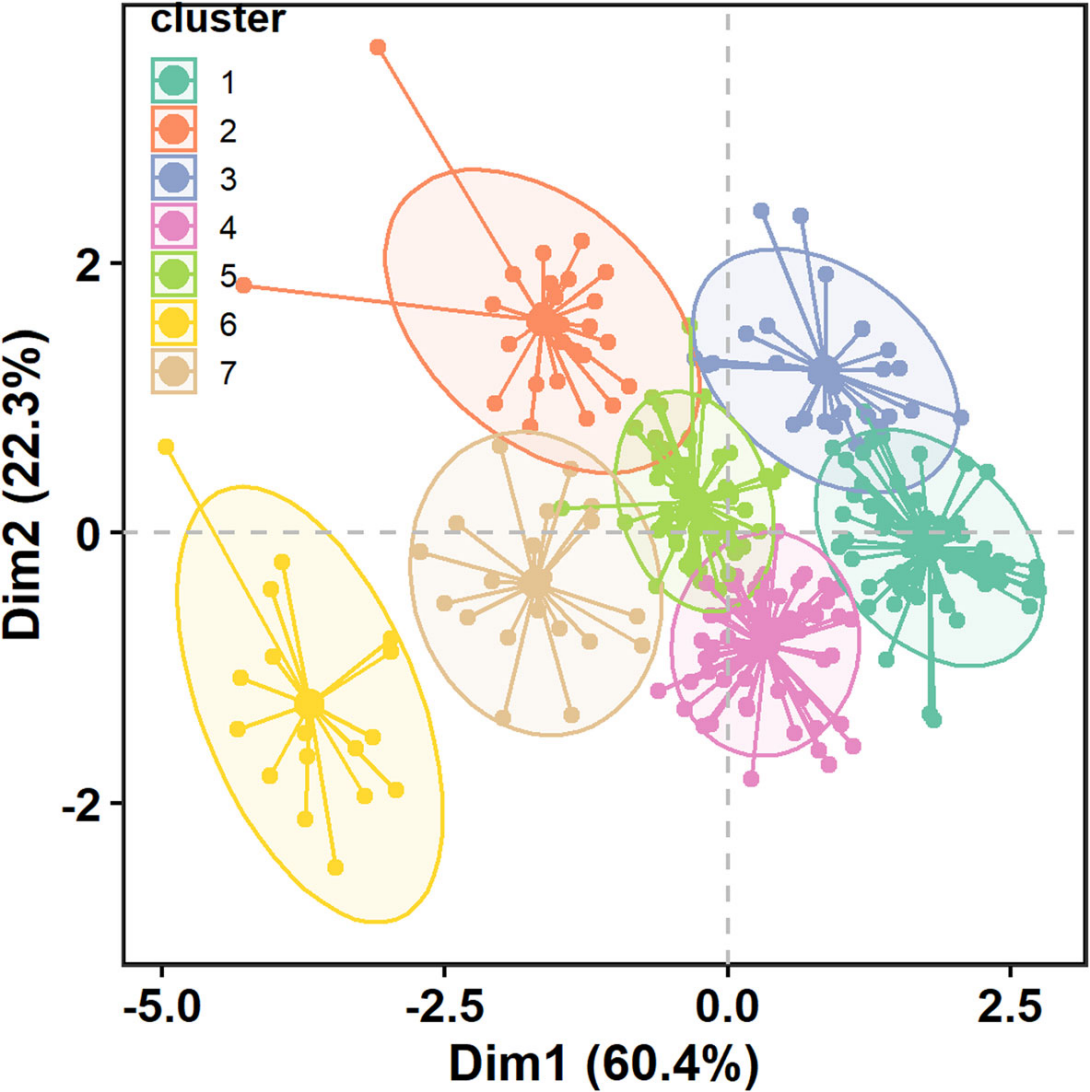
*K*-means partitioning clustering plot of public forest parcels. Each cluster is grouped with parcels with high inter-class similarity. Circular points with the same color belong to the same cluster. Solid lines represent distance from cluster centroid. Ellipses identified a 95% confidence band for each forest parcel cluster

Using the gap statistic iterative method, an optimal number of seven clusters of forest parcels were identified, corresponding to a first maximum *k* statistic value of 0.7 (±0.014) (Fig. 8 in the Appendix). Overall, the first and second dimensions explain 82.7% of the variability of the forest parcels stand, soil, and topographic characteristics (Fig. 3). The first dimension (Dim1) accounted for 60.4% of the forest parcels variability, more than twice the second dimension (Dim2), which explains 22.3% of the variability.

Three of the four variables significantly participate in both dimensions with a percentage contribution over the expected value of 25% (Fig. 9 in the Appendix). The first dimension significantly contributed to both SOC and altitude variables, whereas the second dimension contributed significantly to the MAI. Therefore, the identified forest parcels cluster horizontally placed along the first dimension are significantly different in values of SOC and altitude, whereas those vertically distributed along the second dimension mainly differ in the MAI values.

**Fig. 4:**
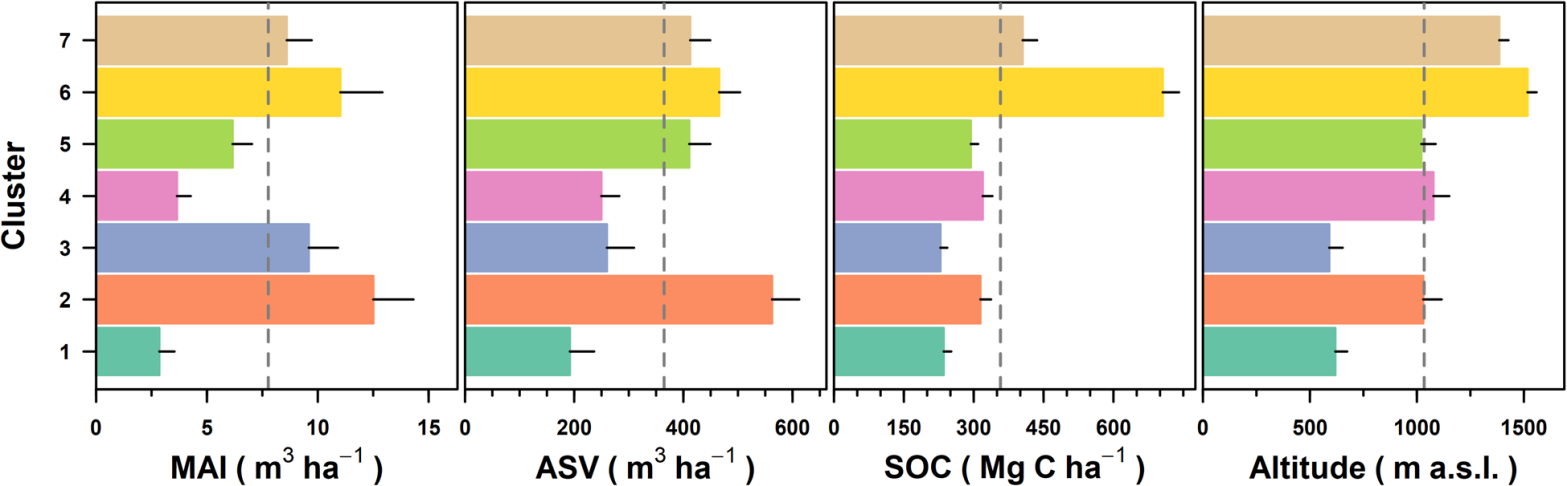
The average value of the mean annual increment (MAI, m^3^ ha^−1^), aboveground standing volume (ASV, m^3^ ha^−1^), soil organic content (SOC, Mg ha^−1^), and altitude (m a.s.l.) for each cluster of the public forest parcels. The colors of horizontal bars represent the K-means partitioning clustering plots (see Fig. 3). Dotted gray vertical lines correspond to the mean values of each forest attribute. The horizontal solid black segments represent the 1st standard deviations

Average value of forest attributes consistently differed among the cluster of public forest parcels (Fig. 4). At the regional scale, the mean value of the MAI was 7.77 (±3.67) m^3^ ha^−1^. At the parcel cluster scale, the average MAI exhibited a broad range of values, from a minimum of 2.85 m^3^ ha^−1^ to a maximum value of 12.51 m^3^ ha^−1^. ASV exhibited an average value at regional scale equal to 364.7 (±134.0) m^3^ ha^−1^, with the high mean value of 526.5 (±99.7) observed for the parcels of cluster 2. Approximately 70% of the clusters of parcels showed a mean value of SOC lower than average values of 357.5 (±164.6) Mg C ha^−1^. Additionally, the clusters with high SOC are located at altitudes markedly above the 1000 m a.s*.*l.

### Scenarios of forest residues release

**Fig. 5:**
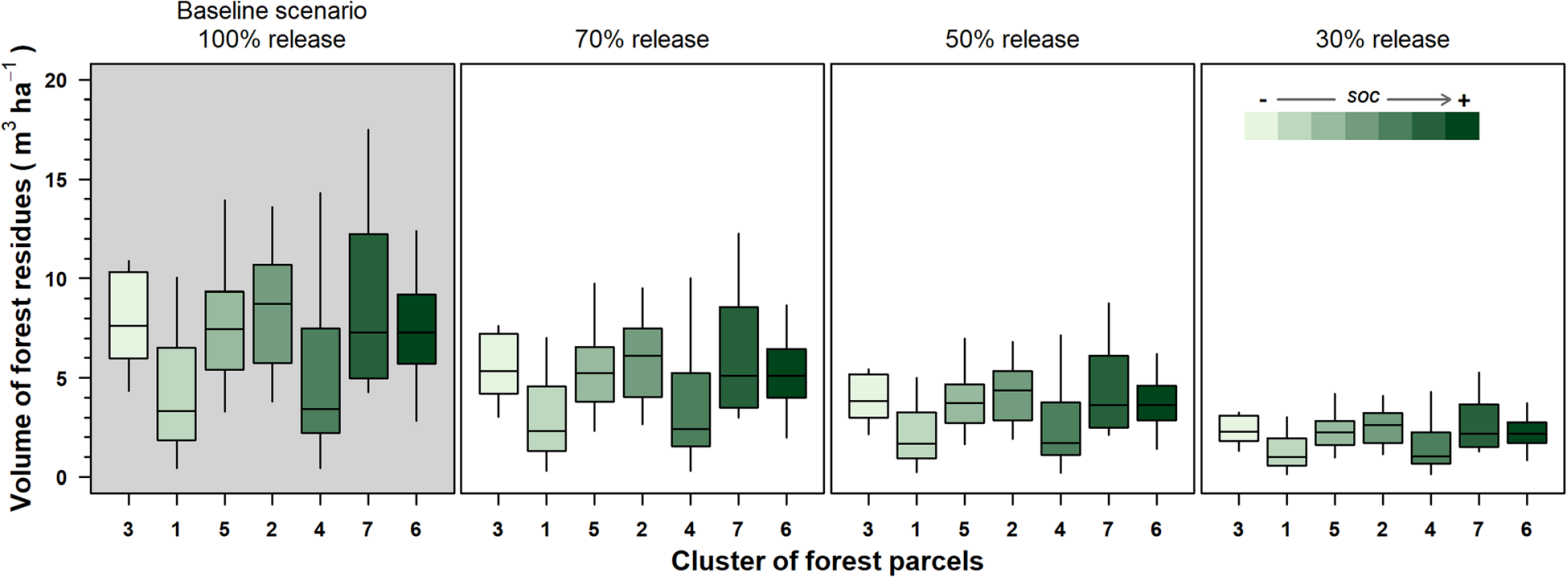
Boxplots of the volume forest residues (m^3^ ha^−1^) for each of the four release scenarios. In the baseline scenario, 100% of forest residues are released upon the forest floor of the logging site (forest parcel). In the three decreasing scenarios, the volume of residues released progressively decreases from 70%, 50%, and 30%. Boxplots are ordered by increasing values of soil organic carbon (SOC) of the clusters of forest parcels, from light green to dark green colors

The volume of the residues changed in line with the clusters of the forest parcels (Fig. 5). In the baseline scenario, available residues ranged from a minimum median value of 3.3 m^3^ ha^*−*1^ in cluster 1 to a maximum of 8.7 m^3^ ha^*−*1^ in cluster 2. This range decreased proportionally with the various scenarios (Fig. 5). In each cluster of parcels of the conservative scenario 30%, the minimum releasable median values of forest residue volumes range from 1.0 m^3^ ha^*−*1^ of cluster 1 and 4 to 2.6 m^3^ ha^*−*1^ of cluster 2. Within these ranges, the variability of forest residues was high and not consistent with the SOC.

### Harvestable forest residues and their carbon content

**Fig. 6:**
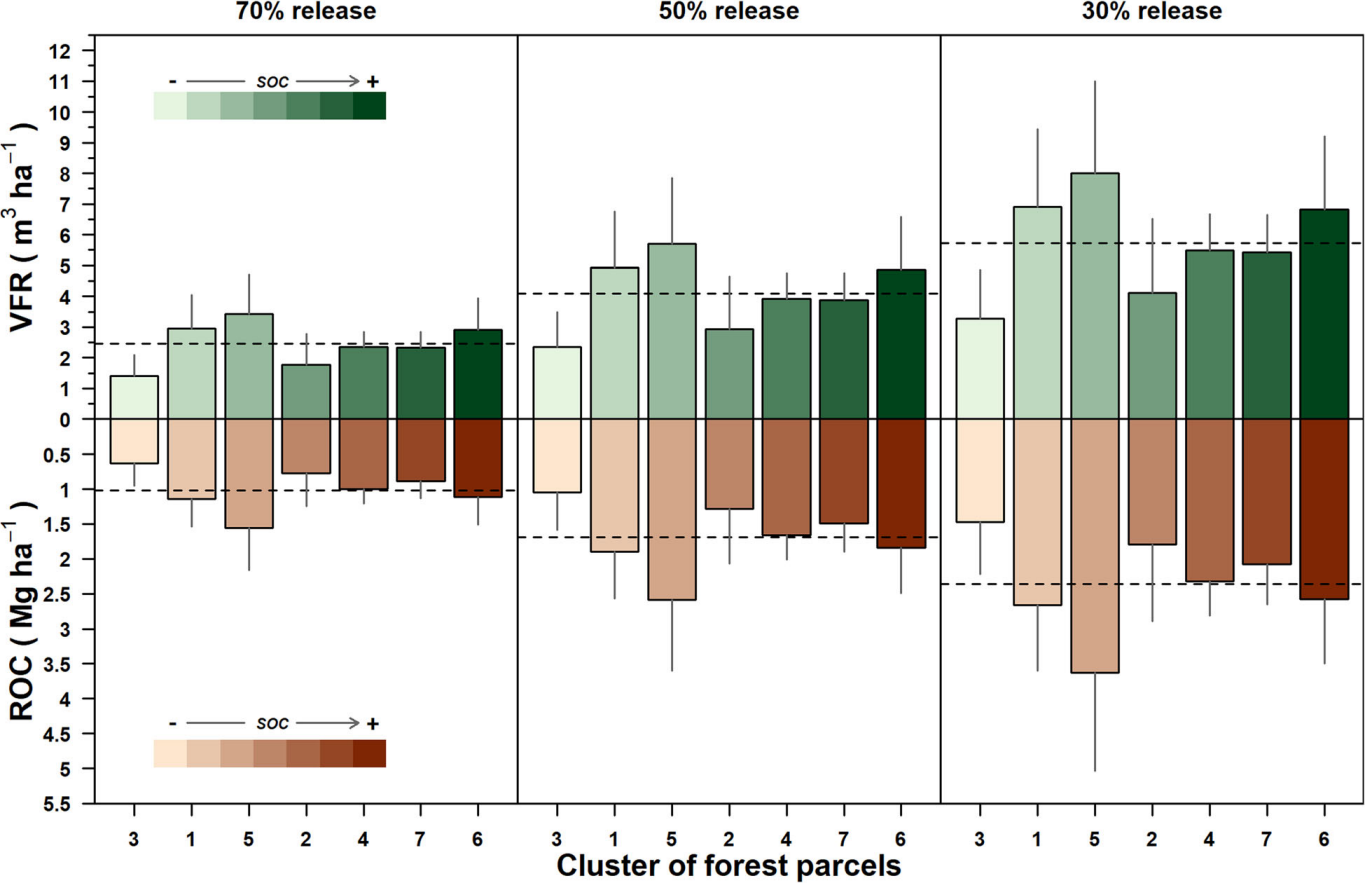
Harvestable volume (VFR, m^3^ ha^−1^) and corresponding carbon content (ROC, Mg C ha^−1^) of forest residues for each of the three decreasing release scenarios: 70%, 50%, and 30%. Black dotted lines represent average values of both VRF and ROC for each scenario. Bars are ordered by increasing values of soil organic carbon (SOC) of the clusters of forest parcels, from light green to dark green colors. In each bar, the vertical gray lines represent the 1st standard deviation

At the regional scale, average harvestable forest residue volumes varied from a minimum of 2.45 (±0.71) m^3^ ha^*−*1^ in the scenario of 70% of on-site release to a maximum of 5.72 (±1.66) m^3^ ha^*−*1^ in the 30% release scenario (Fig. 6). Consequently, the removable organic C of residues varied proportionally to the scenario of the harvestable volumes from 0.90 (±0.26) Mg ha^*−*1^ to 2.11 (±0.61) Mg ha^*−*1^.

Nevertheless, in each scenario, the variability of the harvestable forest residues consistently changed with the changing clusters of parcels. In the release scenario 70%, the harvestable volume of residues ranged between a minimum of 1.40 (±1.37) m^3^ ha^*−*1^ to a maximum of 3.43 (±2.57) m^3^ ha^*−*1^. Such range proportionally broadened with a percentage decrease of forest residues, showing the widest range in the scenario 30%, in which the minimum harvestable volume was 3.27 (±3.20) m^3^ ha^*−*1^ whereas the maximum was 8.00 (±5.99) m^3^ ha^*−*1^.

The potentially removable ROC followed the same pattern as the harvestable volumes (Fig. 6). Indeed, as in the case of forest residue volumes, in each scenario, the ROC was higher for the cluster of parcels 1 and 5, in which SOC was low.

### Sustainability of forest residues removal

**Fig. 7:**
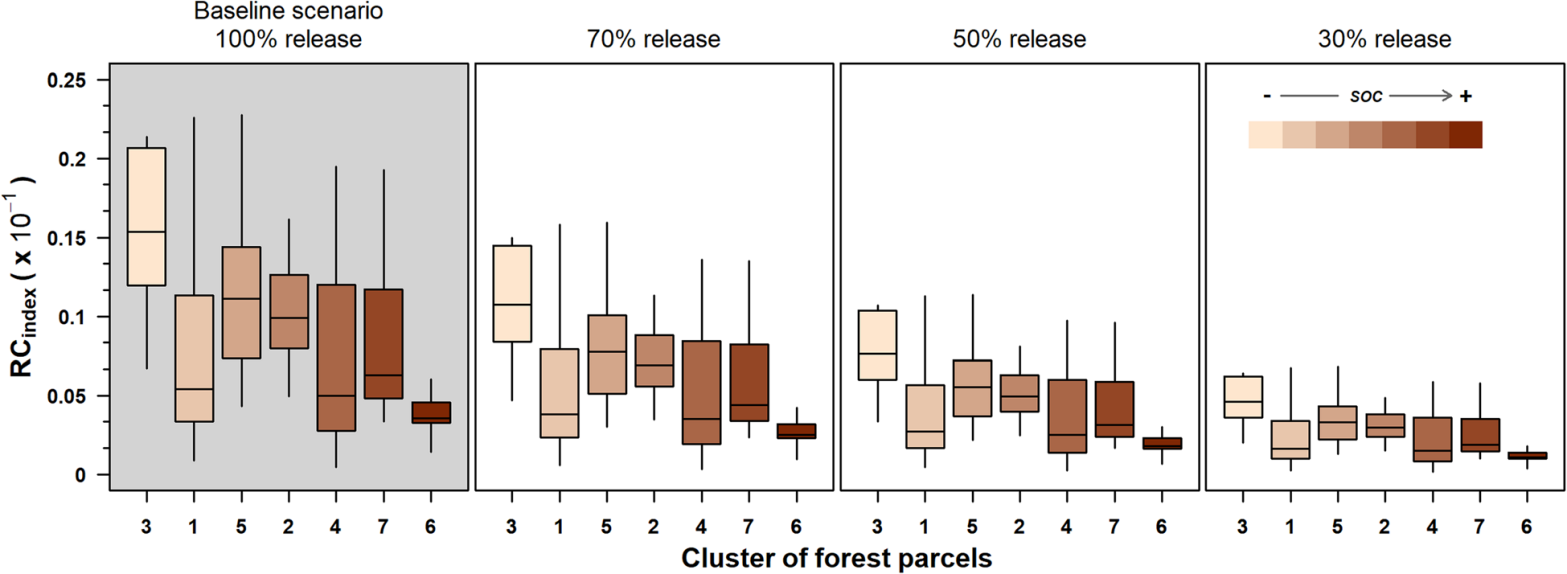
Boxplots of the residual carbon index (RC_index_ 10^−1^) for each of the four forest residues release scenarios. In the baseline scenario, 100% of forest residues are released upon the forest floor of the logging site (forest parcel). In the three decreasing scenarios, the volume of residues released progressively decrease from 70%, 50%, and 30%. Boxplots are ordered by increasing value of soil organic carbon stock (SOCS)

**Fig. 8:**
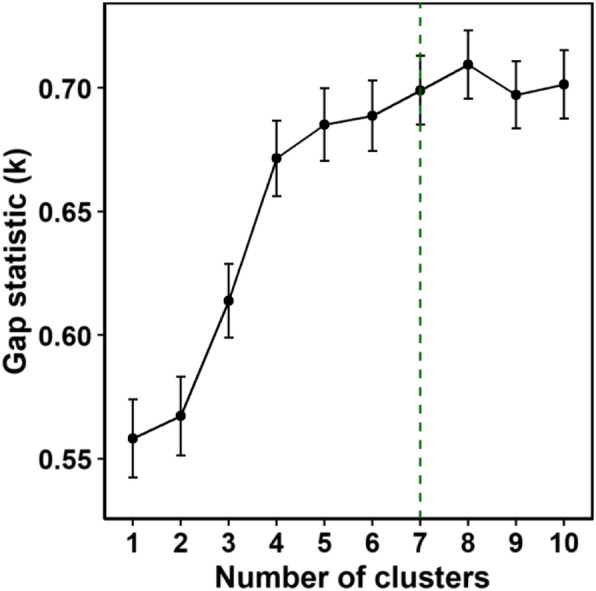
Gap statistic (*k*) curve for standardized attributes of the public forest parcels of the Basilicata region. Black points represent k statistics and whiskers the corresponding standard error (SE). Dotted green line indicates the optimal number of clusters according to “firstSEmax” criteria with a SE factor equal to 1

**Fig. 9:**
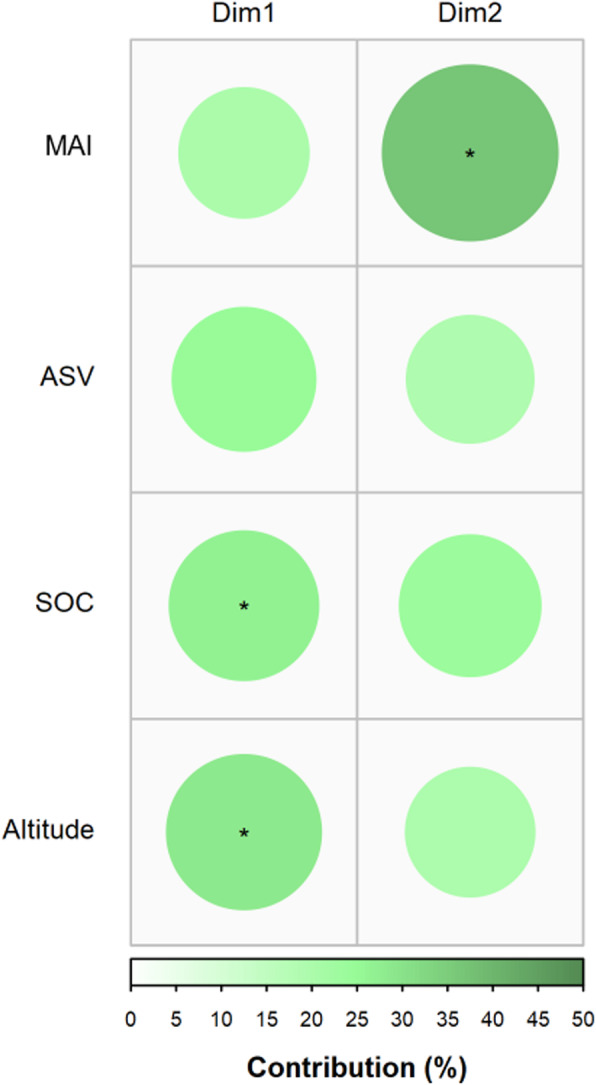
Percentage contribution of the stand (mean annual increment - MAI, aboveground standing volume - ASV), soil (soil organic carbon stocks - SOC), and topographic (Altitude) variables on both first (Dim1) and second (Dim2) components of K-means clustering plots of forest parcels. The symbol * indicates variables with significant contribution over the expected uniform contribution of 25%, on both first and second dimension

Overall, the CR_Index_ of the forest residues potentially releasable on the forest floor range from a maximum value lower than 0.23·10^*−*1^ in the baseline scenario to minimum values of below 0.07·10^−1^ in the 30% release scenario. This ratio decreased with an increase in the SOC of the forest parcel clusters (Fig. 7). In the baseline scenario, the median values of CR_Index_ ranged from 0.01 10^−1^ to 0.08 10^−1^, with the maximum value observed in the forest parcel cluster 1, where SOC is minimum, and a minimum value in cluster 6, which exhibited the highest content of SOC (Fig. 7). The CR_Index_ decreased proportionally with the change in scenario residue release of 70%, 50%, and 30% (Fig. 7). In the cluster of parcels of the scenario 30%, the minimum CR_index_ values vary from a minimum of 0.01·10^−1^ in cluster 4 to a maximum of 0.06·10^−1^ in cluster 3.

## Discussion

The availability of forest residue volume has been assessed by analyzing FMPs, suggesting that approximately 5800 m^3^ year^−1^ (0.66 m^3^ ha^−1^ year^−1^) of forest residue volume could be potentially utilized for bio-economy purposes in the next 10 years. Over the last two decades, different studies have assessed biomass availability obtained from different agricultural and forestry activities, both in northern (Nonini and Fiala 2021) and in southern Italy, especially for bioenergy purposes (Cardinale et al. 2006; Motola et al. 2009). Recently, the estimated potential biomass availability resulting from the management of forest areas in the regional territory accounted for 225,500 tons of fresh woodchips (Cozzi et al. 2013). Our study focused exclusively on the availability of primary forest residues and simulated a set of release scenarios of such residues on logging forest sites as a sustainable practice to counteract soil degradation, especially for public forest stands with low soil organic matter.

On a large scale, the assessment of forest biomass residues requires an appropriate methodological approach (allometric equations or expansion factors) in order to avoid obtaining biased estimates (Somogyi et al. 2007). Nevertheless, in our study, the unavailability of detailed data and their heterogeneity causes uncertainty in the application of the expansion factors. Although the expansion factors cannot be applied, the application of the percentage values reported by Cozzi et al. (2013) realistically estimates the volume residue and their C content. When data are unavailable or unrepresentative, information from default values to approximate local conditions should be considered (IPCC, 2003). However, the application of expansion factor default values could contribute to biased estimation of aboveground biomass residues (Somogyi et al. 2007; Njana 2017).

We evaluated the sustainability of the harvested forest residues by pointing out the trade-off between the removal of biomass residues and their potential impact on the SOC pool of the forests. The SOC content of each parcel was involved in different residue release scenarios, relating them to the C content of the harvested residue volumes left on the forest floor. The high variability observed in SOC and ROC at the forest parcels scale suggests that the management of the release of forest residues should consider the current SOC content and potential input of C from residues to the soil C pool. Accordingly, we suggested modulating the amount of residues releasable on the logging site on the ratio between the potential C fraction of the residues returned to the soil and the C content of the forest soil.

The effects of removing the residues have shown contrasting results, ranging from a considerable decrease to no effect on the soil C stocks (Cozzi et al. 2013). Additionally, consistent levels of biogenic CO_2_ are emitted when the residues are left exposed due to heterotrophic decomposition processes (Cozzi et al. 2013). However, currently, the optimum amount of forest residues potentially incorporable in the organic soil C pool cannot be predicted. Since the SOC is associated with the nutrient availability in soil (related to cation exchange capacity), reducing the C input to the soil in addition to the conventional stem-only harvesting has the potential to deplete the soil C pool and compromise the fertility of forest soil (Achat et al. 2015). Despite this, further research is needed to better understand the contribution of C release from forest residues and how their removal impacts soil degradation in Mediterranean mountain environments.

However, by altering the C input to the soil, the management activities of residues can influence the near- and long-term soil C stocks in forests. These residues, mainly composed of small branches and stem shreds, are usually left on the forest floor due to unfavorable economic circumstances (Sahoo et al. 2019) or burnt on site. In agreement with these standard practices, we define a baseline scenario in which forest residues are left on the forest floor to decompose, simulating a set of precautionary scenarios with a minimum conservative release of 30% of the total forest residues. At a global scale, all forest biomass management guidelines advise leaving a minimum amount of residues on site, although they vary markedly according to forest physiognomies and size of forest residues (Titus et al. 2021). Conventionally, for forest sites where harvested residues can be utilized, guidelines suggest retaining a minimum percentage of residues around 20–30% of their total harvestable amount (Titus et al. 2021). In this context, the simulated release scenarios of 50% and 70% agree with the restrictive guidelines of European countries, which recommend leaving 50–66% of harvest residues on site (Forestry Commission 2009).

In addition, relevant concerns have been expressed about the effects of forestry residue extraction on soil fertility because their removal is associated with the depletion of macro- and micro-nutrients. Such depletion depends on the removed fraction types of tree biomass (Lattimore et al. 2009; Ponder et al. 2012; Achat et al. 2015). In broadleaved deciduous stands, harvesting forest residues mainly originates from the branch and stem fractions, which are both characterized by consistent concentrations of micro- (iron and sodium) and macro-nutrients (potassium, calcium, and phosphorus), especially in their respective bark fractions (Achat et al. 2015). From this perspective, although aboveground biomass content in macro- and micro-nutrients is both site- and species-specific, the degree to which such branches and stem residues are left in situ firmly determines the level of impact of biomass extraction on forest soil mineral nutrients (European Environment Agency 2006).

In recent years, the biomass demand for novel materials (biochemicals) has received more attention as compared to energy usage. Available biomass residues could alternatively be used more efficiently than energy usage (Braghiroli and Passarini 2020). Although branches, tree tops, and bark make up the heterogeneous forest residues from forest operations, several technological approaches have been developed to convert them into wood-based composite panels. Surfactants and solvents currently find essential bio-based applications, although these applications are also gradually shifting toward biomass residuals composting (Bout et al. 2019). These technological advances allow for the value addition to biomass residue and reconsideration of their use toward greater efficiency and environmental compatibility. From a bioenergy perspective, investigating the availability and quantity of residual forest woody materials helps to understand the feasibility of cogeneration or trigeneration plants powered by biomass, which could integrate the production of thermal and electrical energy mix rather than create district energy systems (Fuchs et al. 2016). In this context, the high annual variability in demand for roundwood, the necessity of substantial investments, and the high cost of transportation significantly reduce the companies' gross operating margins, making their use disadvantageous (Pergola et al. 2020). The possibility of integrating profitability with the additional economic value from these residues could represent a significant incentive to support forestry sector investments, which is currently in decline in many Mediterranean areas. Identifying sustainable actions aimed at revitalizing and increasing the multifunctionality of the forest sector, such as developing the bio-based sector, could provide value to forest residues that are underutilized, becoming an opportunity for local socio-economic development.

## Conclusions

The availability of forest residues was investigated in the public forest land of the Basilicata region. Because extracting forest residues to obtain bio-based products can generate environmental impacts, especially reduced soil fertility, this study aimed to minimize such impacts by simulating the release of forest residues on logging sites according to the organic C content of forest parcel, using a scenario analysis compared with a baseline. Although the inventoried forest stands accounted for 2.2% of the total forests of the Basilicata region, the analysis of FMPs represents a pilot study on sustainably managing the forest residues with minimum effect on soil fertility. In the next 10 years, the estimated availability of 5800 m^3^ year^−1^ of biomass residues could represent an integrative resource in the production of the energy mix and a suitable alternative for the bio-based industrial sector. However, managing the harvesting of forest residues according to their potential contributes to the content of C in the soil, which represents a criterion for the sustainable management of soil nutrients and C pools.

## Data Availability

The datasets generated during and/or analyzed during the current study are available from the corresponding author on reasonable request.
